# Associations of Macronutrient Intake Determined by Point-of-Care Human Milk Analysis with Brain Development among very Preterm Infants

**DOI:** 10.3390/children9070969

**Published:** 2022-06-29

**Authors:** Katherine A. Bell, Sara Cherkerzian, Kaitlin Drouin, Lillian G. Matthews, Terrie E. Inder, Anna K. Prohl, Simon K. Warfield, Mandy Brown Belfort

**Affiliations:** 1 Department of Pediatric Newborn Medicine, Brigham & Women’s Hospital, Boston, MA 02115, USA; scherkerzian@bwh.harvard.edu (S.C.); kdrouin@bwh.harvard.edu (K.D.); gabrafam.lillian@gmail.com (L.G.M.); tinder@bwh.harvard.edu (T.E.I.); mbelfort@bwh.harvard.edu (M.B.B.); 2 Harvard Medical School, Boston, MA 02115, USA; akprohl@gmail.com (A.K.P.); simon.warfield@childrens.harvard.edu (S.K.W.); 3 Computational Radiology Laboratory, Department of Radiology, Boston Children’s Hospital, Boston, MA 02115, USA

**Keywords:** human milk analysis, preterm infant, macronutrient, brain volumes, diffusion tensor imaging

## Abstract

Point-of-care human milk analysis is now feasible in the neonatal intensive care unit (NICU) and allows accurate measurement of macronutrient delivery. Higher macronutrient intakes over this period may promote brain growth and development. In a prospective, observational study of 55 infants born at <32 weeks’ gestation, we used a mid-infrared spectroscopy-based human milk analyzer to measure the macronutrient content in repeated samples of human milk over the NICU hospitalization. We calculated daily nutrient intakes from unfortified milk and assigned infants to quintiles based on median intakes over the hospitalization. Infants underwent brain magnetic resonance imaging at term equivalent age to quantify total and regional brain volumes and fractional anisotropy of white matter tracts. Infants in the highest quintile of energy intake from milk, as compared with the lower four quintiles, had larger total brain volume (31 cc, 95% confidence interval [CI]: 5, 56), cortical gray matter (15 cc, 95%CI: 1, 30), and white matter volume (23 cc, 95%CI: 12, 33). Higher protein intake was associated with larger total brain (36 cc, 95%CI: 7, 65), cortical gray matter (22 cc, 95%CI: 6, 38) and deep gray matter (1 cc, 95%CI: 0.1, 3) volumes. These findings suggest innovative strategies to close nutrient delivery gaps in the NICU may promote brain growth for preterm infants.

## 1. Introduction

Preterm birth interrupts a critical period for brain development. During the few months between very preterm birth and term equivalent age—which preterm infants spend in the neonatal intensive care unit (NICU)—the brain’s volume triples, its surface area increases exponentially as gyri and sulci form, and neurons proliferate, migrate, form synaptic connections, and begin myelinating [1,2,3,4]. An adequate supply of nutrients is essential to support these developmental processes, whereas undernutrition can permanently alter brain development with adverse consequences for long-term neurodevelopmental outcomes [5]. Thus, strategies to optimize nutrient delivery in the NICU are essential to support optimal brain growth and structural development, and ultimately to improve long-term outcomes.

Very preterm infants born before 32 weeks’ gestation are vulnerable to both undernutrition and impaired neurodevelopment [6,7]. The current standard of nutritional care for these infants is a human milk diet, based on the established health benefits of human milk, with fortification added to meet this population’s high nutrient needs [8]. Standard fortification—adding a fixed quantity of multicomponent fortifier based on average reference values for macronutrient content—is practiced routinely, but this strategy does not achieve target nutrient intakes for all infants [9]. Due to variability in actual human milk content, up to half of very preterm infants receiving standard fortification do not meet recommended intakes of protein and/or energy [10,11]. These findings point to gaps in macronutrient delivery with standard fortification, with potential adverse consequences for brain growth and development.

Until recently, accurate assessment of the nutrient content of human milk was limited in clinical settings because classical wet laboratory techniques require large milk volumes and are time-consuming and expensive [12]. The advent of commercially available devices that use infrared spectroscopy to analyze small milk volumes at the point-of-care overcomes these obstacles, making human milk analysis clinically feasible in the NICU [12]. Emerging research is investigating the potential of this technology to improve outcomes for preterm infants [9]. The underlying premise is that knowledge about actual milk composition can eliminate gaps in macronutrient delivery, thereby reducing undernutrition and its adverse consequences, including neurodevelopmental impairment. A key component of this premise is that macronutrient intake in the NICU impacts brain development. However, the relationship between macronutrient intake and brain development among preterm infants in the NICU remains uncertain. Prior studies were limited by using average reference values for milk composition rather than direct milk analysis [13,14,15,16,17], which is a substantial limitation given the extent of true variation in macronutrient composition and intakes [18].

Thus, our aim was to determine, among very preterm infants receiving standard fortification, the extent to which directly measured macronutrient intake from human milk during the NICU hospitalization is associated with brain development at term equivalent age. Term equivalent brain magnetic resonance imaging (MRI) allows early assessment of nutrition-sensitive aspects of brain growth and development, prior to the potential confounding influences of the post-discharge environment. Furthermore, MRI measures at term equivalent are predictive of later neurodevelopmental outcomes [14,19,20,21,22]. In this study, we used term equivalent MRI, specifically brain volumetry and fractional anisotropy of white matter tracts, to quantify brain volume and microstructural maturation, respectively. We hypothesized that greater protein and energy intakes would be associated with larger brain volume and more mature white matter microstructure.

## 2. Materials and Methods

### 2.1. Participants

**Figure 1:**
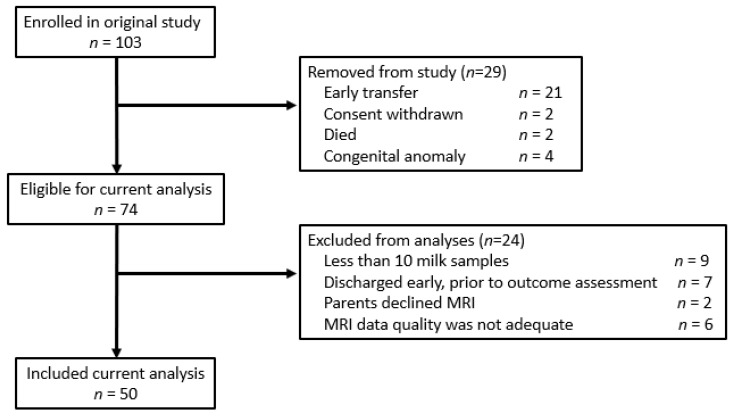
Flow diagram of participants.

We conducted a prospective observational study at an academic level III NICU from 2015 to 2018. Inclusion criteria for enrollment included gestational age <32 weeks, singleton or twin, and maternal intention to provide breastmilk for her infant(s). Infants with major congenital anomalies were excluded. Of the 103 infants initially enrolled, 29 were removed from the study due to subsequent diagnosis of congenital anomaly (*n* = 4), death (*n* = 2), parent withdrawal of consent (*n* = 2), or transfer to another hospital shortly after enrollment (*n* = 21). Of the remaining 74 infants, for this analysis we included 50 who had at least 10 breastmilk samples and research-quality brain MRI data available (Figure 1). Routine clinical care included parenteral nutrition for all infants with birthweight <1800 g, advancement of enteral feeding to goal 150–160 mL/kg/day, standard milk fortification with multicomponent bovine-based human milk fortifier, and addition of liquid protein and/or medium chain triglycerides as indicated by growth faltering (clinical practice guideline available upon request). The study was approved by the Partners Human Research Committee (protocol number 2014P001834) and parents of all participating infants gave written informed consent.

### 2.2. Human Milk Analysis

As previously reported [18], we collected 5 mL samples of unfortified maternal breastmilk once daily on weekdays, starting from the date that infants reached 100 mL/kg/day of enteral feeds and continuing through 36 weeks’ postmenstrual age. To best represent the infant’s actual diet, we intentionally sampled the milk being fed to the infant that day, including milk pooled from one or more pumping sessions; some or all of the milk may have been previously frozen. After gently mixing and sampling the unfortified milk, bedside nurses fortified the remaining milk per clinical order in preparation for feeding the infant. The 5 mL sample was stored at 4 °C while awaiting analysis, which was performed later the same day.

We homogenized and analyzed milk samples once using a point-of-care human milk analyzer that employs mid-infrared spectroscopy (Miris AB, Uppsala, Sweden) according to the manufacturer instructions [23]. This device estimates the milk sample’s protein, fat, and carbohydrate content in g/100 mL, and calculates energy content (kcal/100 mL) from the macronutrient values. The high accuracy and reliability of the device, both in general and for the specific device in our unit, have been reported [18,24].

### 2.3. Brain Magnetic Resonance Imaging

At term equivalent age, infants underwent brain MRI without sedation [25] using a Siemens Trio 3 Tesla scanner (Siemens AG, Erlangen, Germany). T_2_-weighted images were acquired with a sagittal T2 turbo spin echo sequence, with 1 mm isotropic voxels, flip angle = 160°, repetition time = 8630 ms, echo time = 133 ms, FOV = 190 × 190 mm, matrix = 192 × 192. We used an automated segmentation method (Morphologically Adaptive Neonatal Tissue Segmentation [26]) to calculate total brain volume and tissue-specific volumes of the cortical gray matter, deep gray matter, white matter, hippocampus, and cerebellum.

Diffusion-weighted images were acquired with 30 directions at *b* = 1000 s/mm^2^ with 1 *b* = 0, and 2 mm isotropic voxels. To analyze the diffusion-weighted images, we first examined the MRI images and removed diffusion-weighted volumes with artifact prior to analysis. Scans with >50% of volumes removed (*n* = 5) were excluded. All processing and analyses were completed using the Computational Radiology Kit (http://crl.med.harvard.edu, accessed on 14 March 2019) via a fully automated processing pipeline [27,28,29]. We defined 15 regions of interest using a validated fully automatic method [30] and validated labelling schemes for white matter tractography [31,32]. The 15 regions were the corpus callosum and bilateral anterior thalamic radiations, cingula, corticospinal tracts, inferior longitudinal fasciculi, optic radiations, posterior limb of the internal capsule (PLIC), and uncinate fasciculi. We computed mean fractional anisotropy (FA) within each tract, considering the right and left tracts separately.

Eight infants lacked volumetric data, and one lacked diffusion data, due to unavailability of research-quality data or inability to process the imaging data.

### 2.4. Clinical Data

We used the electronic medical record to abstract the daily volume of milk fed to the infant, percent of feed comprising maternal milk (as compared with donor human milk or formula), daily weight measured to 1 g on calibrated digital infant scales (Scale-Tronix, Inc., White Plains, NY, USA), demographics including gestational age, sex, and birthweight, and common comorbidities of preterm birth including necrotizing enterocolitis Bell stage ≥2 (NEC), culture-proven sepsis, respiratory support at 36 weeks, patent ductus arteriosus treatment, and intraventricular hemorrhage according to standard definitions [33]. Birthweight Z-score was calculated from the Fenton reference [34].

### 2.5. Data Analysis

Prior to calculating the exposure variables of median macronutrient intakes, we excluded extreme macronutrient values, defined as > 3 standard deviations from the mean of all samples in our cohort, as likely to represent measurement errors (*n* = 103 samples excluded, from 1878 samples collected).

The primary outcomes were measures of brain size and maturation, namely total and tissue-specific brain volumes, and fractional anisotropy in the 15 white matter tracts. The primary exposures were macronutrient and energy intakes, which we categorized into quintiles because the distribution of nutrient intakes was skewed rather than normally distributed. To determine the exposure variables, we first calculated daily intake of individual macronutrients (in g/kg/day) and energy (kcal/kg/day) by multiplying measured milk content (g/mL) by the volume of milk ingested (mL) and dividing by the infant’s weight (kg) that day. Then, we summarized macronutrient intake for each infant by calculating the median intake of protein, fat, carbohydrate, and energy from all milk samples fed to that infant throughout the study period. Finally, we assigned infants to quintiles of macronutrient intake based on the distribution of nutrient intake in our cohort. Quintile assignments were made independently for each macronutrient. For our analyses, we dichotomized the quintiles into the top quintile (>80 percentile, quintile 5) versus the lower remaining quintiles (≤80 percentile, quintiles 1–4) to test our hypothesis that high levels of macronutrient intake (>80 percentile) are associated with greater brain volume than lower levels (≤80 percentile), based on the scientific premise that the highest intakes would be most biologically impactful.

We used median regression to determine associations between being in the top quintile of nutrient intake and each brain MRI outcome, because linear models violated the requirement for normality of residuals. We included in the model covariates identified a priori as being associated with nutrient intake and brain volume or maturation, namely, gestational age at birth, sex, birthweight Z-score, postmenstrual age at time of MRI, and a composite variable for comorbidities of prematurity that represented having at least one of the following diagnoses known to have negative associations with brain size or development [35,36,37]: NEC, sepsis, patent ductus arteriosus treatment, respiratory support at 36 weeks, and postnatal steroid use. Our model also accounted for non-independence of twins. Analyses were performed using STATA version 17 (StataCorp LLC, College Station, TX, USA).

## 3. Results

The 50 participants had mean gestational age 28.2 weeks (standard deviation [SD] 2.4) and birthweight Z-score −0.2 (SD 1.0). (Table 1) Infants were exclusively or predominantly fed maternal milk through the first month of life; at 28 days old, 46 of 50 infants were receiving exclusively maternal milk, 2 were receiving ≥75% maternal milk, and 2 were receiving ≥50% donor milk but had received exclusively human milk through 21 days old.

We analyzed a total of 1775 breastmilk samples. The mean number of samples per infant was 38 (SD 17.1). Infants were categorized into quintiles of macronutrient intake as described above; the mean and range of nutrient intake in each quintile is in Table 2.

The adjusted associations of nutrient intakes with brain volumes are shown in Table 3. We found positive associations of energy intake with most brain volumes; 95% confidence intervals excluded the null hypothesis for total brain volume, cortical gray matter, and white matter volume. Specifically, infants in the highest quintile of energy intake, as compared with the lower four quintiles, had an additional 31 cc (95% confidence interval [CI]: 5, 56) larger total brain volume, 15 cc cortical gray matter (95% CI: 1, 30), and 23 cc (95% CI: 12, 33) larger white matter volume. Higher protein intake was associated with larger volumes of the cortical gray matter (22 cc [95% CI: 6, 38]), deep gray matter (1 cc [95% CI: 0.1, 3]), and total brain (36 cc [95% CI: 7, 65]). The estimated associations between fat or carbohydrate intake and brain volumes demonstrated wide confidence intervals that did not exclude the null.

Table 4 shows the adjusted associations of nutrient intakes with fractional anisotropy of white matter tracts. While the effect estimates were generally positive in most white matter tracts, 95% confidence intervals did not exclude the null.

Estimates represent the difference in brain volume associated with being in the top quintile versus the lower four quintiles of nutrient intake, adjusted using median regression for gestational age at birth, sex, postmenstrual age at time of brain MRI, birthweight Z-score, composite comorbidity variable, and accounting for the non-independence of infants born to the same mother.

## 4. Discussion

In this study, we used point-of-care human milk analysis to accurately determine macronutrient intake from human milk among very preterm infants, so that we could examine associations with measures of brain development. Consistent with our hypothesis, we found that higher intakes of energy and protein were associated with larger total and regional brain volumes at term equivalent age. To our knowledge, ours is the first study to assess directly measured macronutrient intake from human milk in association with quantitative MRI measures of brain size in very preterm infants. Our results provide support for the concept that addressing residual gaps in nutrient delivery among fortified human milk-fed very preterm infants may improve brain development during the critical preterm period.

Our results linking energy intake and brain size are consistent with several [13,14,15]—though not all [16,17,38]—prior studies of macronutrient intake and brain development in preterm infants. Among three prior studies that reported positive associations between macronutrient intake and brain volumes [13,14,15], all found that energy intake was linked to brain size, but varied in the brain region involved, with the most common regions being the cerebellum [13,14,15], basal ganglia [13,14], and total brain volume [14]. In contrast to energy, prior studies are inconsistent with respect to the relationship between protein intake and brain volume; some linked protein intake to the same regions as energy intake [13,14] while others found no relationship between protein intake and brain size [15,16,17]. Our finding that protein and energy intake were associated with cortical gray matter volume is novel is consistent with experimental evidence suggesting that the cortex is particularly sensitive to protein-energy malnutrition [39]. The inconsistency in reported relationships from prior studies, both in terms of the macronutrients and brain regions implicated, may stem in part from a lack of accuracy in the assessment of macronutrient intake due to using average reference values. Our study using directly measured milk content confirmed the link between energy intake and total brain volume, while also demonstrating novel associations of energy intake with white matter volume and of protein and energy intake with cortical gray matter volume. Overall, our findings, taken together with prior reports, support the concept that variations in nutrient intake in the NICU contribute to differences in brain size at term.

Our study also expands the limited prior literature examining the relationship between macronutrient intake and white matter microstructure. Prior studies that used average milk content yielded conflicting findings with respect to both the nutrients implicated and the brain regions affected. Two small studies found positive associations of cumulative fat and energy intake with fractional anisotropy, but conflicted regarding the tract impacted; one found positive associations with the PLIC [13], while the other found associations with five white matter tracts (posterior corona radiata, thalamic radiations, superior longitudinal fasciculus, corticospinal tract, and superior corona radiata) but not the PLIC [14]. In addition, one study in extremely preterm infants found a positive association between higher protein—rather than fat—intake in the first week of life and FA in many of the same tracts as above, including portions of the corona radiata, thalamic radiations, superior longitudinal fasciculus, and internal capsule, plus the corpus callosum [40]. In contrast, we studied many of the same white matter tracts but found no association between macronutrient or energy intake and FA in any of the 15 tracts assessed. Noting that we generally found positive effect estimates, our small sample size may have limited our power to exclude the null, although our sample size is similar to other studies that identified positive relationships [14]. Alternatively, other factors known to impact white matter microstructural maturation, and which we did not measure in this study, such as inflammation, brain injury, and perinatal exposures [41], may overshadow any independent effect of nutrient intake on white matter development.

Macronutrients may influence brain growth and development through multifactorial pathways. Individual macronutrients play specific roles in brain development, beyond providing energy to support the energy demands of brain growth [42], and may additionally be neurorestorative after brain injury [43]. Protein is required for the production of growth factors, structural proteins, and myelin [39]. Carbohydrates provide lactose in addition to non-digestible bioactive human milk oligosaccharides. Fat, particularly long-chain polyunsaturated fats, is incorporated into neuronal membranes and myelin [39]. While several prior studies suggested that fat intake has an independent effect on brain size [13,14,15], our study found no such association. In contrast, our results using directly measured macronutrient content suggest that while higher energy intake does predict larger brain size, variations in protein intake—rather than fat—from human milk may have an independent effect on brain size. Thus, measuring protein intake specifically, in addition to total energy intake, may be important when designing nutritional strategies that promote brain growth.

Our results have important implications for the development of innovative clinical strategies that incorporate point-of-care human milk analysis to ensure adequate nutrient delivery for very preterm infants. In this study, we found that even under conditions of routine standard fortification, variability in nutrient intake from human milk has a clinically detectable association with structural brain development. These findings support the concept that eliminating existing gaps in nutrient delivery stemming from variability in human milk content may support improved brain development among very preterm infants. One emerging strategy utilizing point-of-care human milk analysis is individually targeted fortification, in which repeated measurements of milk content are used to individualize fortification for each infant, ensuring that the goal macronutrient and energy intakes are met [44,45,46]. Several small trials using this strategy for very preterm or very low birthweight (VLBW) infants have demonstrated its effectiveness in increasing macronutrient intake and improving weight gain [10,47,48]. For example, a recent randomized trial found that infants who received targeted fortification had on average greater intakes of fat by 0.5 g/kg/day, carbohydrates by 2.8 g/kg/day, protein 0.9 g/kg/day, and energy 20 kcal/kg/day [10]. If infants from the lowest quintile in our cohort had received these additional intakes, their median intake of protein and energy would have been similar to infants in the highest quintile in the cohort, which was associated with substantially greater brain volume (31 cc, or 0.8 standard deviations of the mean) in our study. While direct evidence for the impact of targeted fortification on neurodevelopment is currently lacking, observational studies demonstrate that higher macronutrient intake is associated with improved neurodevelopment [49], and we expect that ongoing randomized trials [50,51], including our own [51], will further clarify the effects of targeted fortification on structural brain development and neurodevelopmental outcomes. Taken together, our findings combined with other studies linking macronutrient intake to brain growth and neurodevelopmental outcomes provide support for using point-of-care human milk analysis to improve macronutrient delivery as a promising clinical strategy to optimize neurodevelopment.

The strengths of our study include the accurate quantification of daily macronutrient intake through direct analysis of milk utilizing a validated and accurate device for point-of-care human milk analysis. Another strength of our study is the direct quantification of brain volume using MRI, which is more accurate than using proxies such as head circumference, and allows for the identification of regional sensitivities in brain size [52]. While neurodevelopmental testing was not available in our cohort, the brain MRI measures used in our study have been linked to later neurodevelopmental outcomes; specifically, brain volume at term is an independent predictor of cognitive outcomes in early childhood and school age [14,19,20,21,22,53]. The focus of our study was specifically to examine the impact of variability in human milk composition; therefore, we did not include all possible sources of nutrient intake (for example, human milk fortifier or parenteral nutrition). Unlike human milk, the nutrient content of these sources is standardized and thus their contribution to nutrient delivery is accurately known. Furthermore, prior studies show that the use of standard fortification increases nutrient delivery for all infants by the same amount while preserving inter-individual variation stemming from human milk [54]. The sample size of our study may have limited our power to detect small associations between certain nutrients or brain regions, although we had sufficient power to identify associations between energy or protein intake and several specific brain regions. As with all observational studies, our findings could be subject to residual confounding, although we were able to adjust for several factors known to be associated with nutrient intake and brain size, including gestational age and birth weight. Finally, this single center study of very preterm infants may not be generalizable to infants born at later gestational ages or in units utilizing different care practices.

## 5. Conclusions

Our results suggest that improving macronutrient delivery during the critical preterm period could improve concurrent brain growth, with the potential for positive impacts on later neurodevelopment. Point-of-care human milk analysis is now feasible in the neonatal intensive care unit using commercially available regulatory-approved devices. We expect that ongoing randomized trials (including ours [51]) of innovative fortification strategies incorporating human milk analysis will yield further evidence regarding the benefits of this approach to short- and long-term outcomes.

## Figures and Tables

**Table 1:** Participant characteristics (*n* = 50).

Characteristic				
	Number	Percent		
Male	25	50%		
Race				
White	25	50%		
Black	15	30%		
Asian	3	6%		
Other or unknown	7	14%		
Small for gestational age (birthweight < 10%) ^1^	8	16%		
Multiple gestation	15	30%		
Respiratory support at 36 weeks ^2^	21	42%		
Postnatal steroids	8	16%		
Necrotizing enterocolitis (NEC) Bell stage ≥ 2	1	2%		
Culture-proven sepsis	3	6%		
Patent ductus arteriosus treatment	10	20%		
Intraventricular hemorrhage grade 3 ^3^	2	4%		
Combined comorbidity variable ^4^	27	54%		
	Mean	SD	Range	
Gestational age at birth (weeks)	28.2	2.4	23 4/7 to 31 6/7	
Birth weight (g)	1049	399	410 to 2065	
Birth weight Z-score ^1^	−0.2	1.0	−2.4 to 1.9	
Weight Z-score at term equivalent age ^1,5^	−0.8	1.1	−3.9 to 0.6	
Number of milk samples per infant	38.7	17.1	14 to 84	
Postmenstrual age at MRI scan (weeks)	38.8	1.50	35 5/7 to 41 2/7	
Brain volumes (cc) at term equivalent age				
Cortical gray matter	131	26		
Deep gray matter	23	2		
White matter	132	17		
Hippocampus	3	1		
Cerebellum	23	4		
Total brain volume	319	38		

^1^ From Fenton reference charts. ^2^ Includes mechanical ventilation, continuous positive airway pressure (CPAP), high flow and low flow nasal cannula. ^3^ No infants in our cohort had grade 4 intraventricular hemorrhage. ^4^ Includes NEC, sepsis, patent ductus arteriosus treatment, respiratory support at 36 weeks, and postnatal steroid use. ^5^ At time of MRI scan.

**Table 2:** Macronutrient intake from human milk in each quintile.

	Quintile 1 *n* = 10	Quintile 2 *n* = 10	Quintile 3 *n* = 10	Quintile 4 *n* = 10	Quintile 5 *n* = 10
Protein (g/kg/day)	1.1 ± 0.1 (0.9, 1.2)	1.3 ± 0.1 (1.2, 1.3)	1.4 ± 0.02 (1.4, 1.5)	1.6 ± 0.1 (1.5, 1.7)	2.1 ± 0.3 (1.7, 2.6)
Fat (g/kg/day)	3.4 ± 0.2 (3.1, 3.6)	3.8 ± 0.1 (3.7, 4.0)	4.1 ± 0.1 (4.0, 4.3)	4.6 ± 0.2 (4.3, 4.8)	5.3 ± 0.4 (4.8, 6.3)
Carbohydrates (g/kg/day)	8.4 ± 0.8 (6.3, 8.8)	9.0 ± 0.1 (8.8, 9.2)	9.3 ± 0.1 (9.2, 9.4)	9.4 ± 0.1 (9.4, 9.5)	10.4 ± 1.9 (9.5, 15.8)
Energy (kcal/kg/day)	74.8 ± 3.9 (65.2, 79.0)	80.3 ± 0.5 (79.5, 81.3)	83.2 ± 1.3 (81.7, 85.3)	87.7 ± 2.0 (85.5, 90.7)	97.4 ± 10.8 (91.1, 125.6)

Results presented as mean ± standard deviation (range).

**Table 3:** Associations of macronutrient intake with brain volume at term equivalent age (*n* = 42).

	Estimated Additional Brain Volume in cc (95% CI) for Infants in Top Quintile (>80th Percentile) versus All Other Quintiles (≤80th Percentile) of Macronutrient Intake.					
	Total Brain Volume	Cortical Gray Matter	Deep Gray Matter	White Matter	Hippocampus	Cerebellum
Protein	36.0 * (7.1, 64.8) *p* = 0.02	22.2 * (6.7, 37.8) *p* = 0.006	1.5 * (0.1, 2.9) *p* = 0.04	18.8 (−10.0, 47.6) *p* = 0.19	0.4 (−0.4, 1.1) *p* = 0.30	−1.1 (−3.6, 1.4) *p* = 0.37
Fat	11.7 (−27.6, 51.0) *p* = 0.55	0.6 (−19.3, 20.6) *p* = 0.95	0.2 (−1.8, 2.2) *p* = 0.85	14.1 (−3.4, 31.6) *p* = 0.11	0.2 (−0.6, 1.1) *p* = 0.60	−1.4 (−3.1, 0.2) *p* = 0.08
Carbohydrate	18.7 (−12.0, 49.5) *p* = 0.23	19.9 (−0.8, 33.3) *p* = 0.06	1.6 (−0.1, 3.2) *p* = 0.06	14.6 (−6.8, 36.1) *p* = 0.17	0.4 (−1.0, 1.7) *p* = 0.52	0.9 (−2.8, 4.6) *p* = 0.62
Energy	30.9 * (5.5, 56.4) *p* = 0.02	15.3 * (0.8, 29.9) *p* = 0.04	1.0 (−1.0, 3.0) *p* = 0.32	22.9 * (12.2, 33.4) *p* < 0.001	−0.1 (−1.1, 0.8) *p* = 0.81	−1.0 (−3.4, 1.5) *p* = 0.42

* *p* < 0.05.

**Table 4:** Associations of macronutrient intake with fractional anisotropy of white matter tracts at term equivalent age (*n* = 44).

	Estimated Increase in Fractional Anisotropy in % (95% CI) for Infants in (>80th Percentile) versus All Other Quintiles (≤80th Percentile) of Macronutrient Intake.														
	Left ATR	Right ATR	CC	Left CI	Right CI	Left CST	Right CST	Left ILF	Right ILF	Left OR	Right OR	Left PLIC	Right PLIC	Left UF	Right UF
Protein	0.3 (−1.7, 1.0)	0.1 (−0.9, 1.1)	0.3 (−1.9, 2.4)	0.7 (−0.4, 1.8)	−0.3 (−2.0, 1.4)	0.2 (−1.1, 1.6)	2.0 (−0.5, 4.4)	−0.2 (−1.7, 1.3)	−0.1 (−1.6, 1.3)	0.3 (−1.4, 2.1)	0.9 (−1.2, 2.9)	0 (−1.7, 1.8)	0.1 (−1.0, 1.3)	−0.6 (−2.1, 0.9)	0 (−0.9, 0.7)
Fat	0.1 (−1.6, 1.8)	0.4 (−1.5, 2.3)	0.7 (−3.1, 4.5)	0.9 (−0.6, 2.3)	0.5 (−2.1, 3.1)	0.9 (−1.3, 3.1)	1.8 (−1.7, 5.2)	1.5 (−0.1, 3.1)	0.3 (−1.0, 1.6)	0.9 (−0.8, 2.6)	1.4 (−0.9, 3.7)	0.5 (−1.0, 2.0)	0.5 (−0.9, 2.0)	1.3 (−0.5, 3.1)	0.5 (−0.6, 1.7)
Carbohydrate	−0.4 (−1.7, 0.8)	0.6 (−0.9, 2.0)	0.3 (−1.7, 2.3)	−0.6 (−1.7, 0.6)	−0.5 (−2.1, 1.0)	0.2 (−1.1, 1.6)	2.0 (−0.5, 4.4)	−0.9 (−2.1, 0.5)	−0.9 (−2.1, 0.4)	−0.4 (−2.1, 1.3)	−0.6 (−2.9, 1.6)	0 (−1.5, 1.5)	−1.0 (−1.5, 1.3)	0.6 (−0.9, 2.0)	0 (−1.0, 0.9)
Energy	0.4 (−1.2, 2.0)	−0.1 (−2.1, 2.0)	−0.9 (−2.6, 0.8)	0.6 (−1.0, 2.2)	−0.3 (−2.4, 1.9)	0.5 (−1.1, 2.2)	1.8 (−0.3, 3.9)	0.5 (−1.8, 2.8)	0.2 (−1.3, 1.7)	0.4 (−1.5, 2.3)	0.5 (−1.5, 2.5)	0 (−1.7, 1.6)	0.3 (−1.2, 1.8)	1.3 (−1.3, 4.0)	0 (−1.2, 1.0)

All *p*-values were >0.05. Estimates represent the difference in fractional anisotropy (in %) associated with being in the top quintile versus the lower four quintiles of nutrient intake, adjusted using median regression for gestational age at birth, sex, postmenstrual age at time of brain MRI, birthweight Z-score, composite comorbidity variable, and accounting for the non-independence of infants born to the same mother. ATR, anterior thalamic radiations; CC, corpus callosum; CI, cingulum; CST, corticospinal tract; ILF, inferior longitudinal fasciculus; OR, optic radiations, PLIC, posterior limb of the internal capsule; UF, uncinate fasciculus.

## Data Availability

The data presented in this study are available upon reasonable request from the corresponding author. The data are not publicly available to maintain privacy of personally identifiable data for the participants.
